# Interactive Game-Based Platform System for Assessing and Improving Posture Control in the Elderly

**DOI:** 10.3390/bioengineering10111291

**Published:** 2023-11-08

**Authors:** Pi-Chang Sun, Chen-Yang Kao, Chung-Lan Kao, Shun-Hwa Wei

**Affiliations:** 1 Department of Rehabilitation Medicine, Taipei City Hospital, Taipei City 11556, Taiwan; 2 School of Medicine, National Yang Ming Chiao Tung University, Taipei City 11221, Taiwan; 3 Department of Physical Therapy and Assistive Technology, National Yang Ming Chiao Tung University, Taipei City 11221, Taiwan; 4 Department of Physical Medicine and Rehabilitation, Taipei Veterans General Hospital, Taipei City 11217, Taiwan

**Keywords:** balance, center of pressure, elderly, interactive training, limit of stability

## Abstract

Inadequate response to balance perturbations lead to posture instability in the elderly. The fall risks are increased by a reduced capacity to control the center of pressure (COP) displacement within the safety limit of the supporting base. This study developed an interactive evaluation and training platform. The system incorporated a computerized program with instantaneous force plate evaluation. Ten young subjects underwent a baseline evaluation and twenty-nine community-dwelling elderly received pre- and post-intervention testing. The ability to reach the stability limit was assessed by measuring the maximum voluntary excursion of the COP in anterior–posterior and medial–lateral directions. Functional mobility tests including Berg Balance Scale, Timed-Up-and-Go and functional reach were used as functional outcomes. The experimental group (*n* = 15) received a 40 min intervention three times a week for six weeks. The interactive game-based training focused on multi-directional weight shifting by tracing a COPcontrolled target to challenge an individual’s stability limit. The control group (*n* = 14) maintained daily activities as usual. The young group revealed a superior COP displacement through active ankle control than the elderly, especially in the anterior–posterior direction. The experimental group improved their COP displacement control more in the medial–lateral direction due to the predominant side-to-side gameplay movement. The functional outcome measures were also significantly improved after training. Using the COPcontrolled game-based program, the stability limit was challenged to facilitate dynamic posture control by an incremental increase in self-initiated perturbations. The platform system could assist in transferring the immediate training effects into daily functional mobility in the elderly.

## 1. Introduction

Postural instability is well known increasing the risk of fall-related injuries in the elderly [1]. The ability to maintain balance control is important for independent functional mobility. Most falls occur during dynamic tasks rather than static conditions, such as functional reach and weight shifting on a non-moving support base in the standing position [2]. People must take corrective actions prior to reaching their stability limit of the base of support (BOS). Older adults have an impaired capacity of the axial body to recover from postural perturbations during usual dynamic activities. These people lose posture steadiness when their centers of mass (COM) fall outside of their BOS. Ensuring a proper dynamic relationship between the COM and the BOS is essential to fall prevention in the elderly [3]. The functional mobility should be enhanced to control the projective direction and momentum of the COM within the support base.

Previous clinical tests were shown to exhibit ceiling effects when predicting fall risks in healthy elderly people [4,5]. Postural control characteristics can be assessed using a computerized force plate by collecting the data from the center of pressure (COP) excursions on the base of support. Most studies asked participants to maintain quiet standing as steadily as possible. However, these force plate parameters revealed varying degrees of spontaneous COP sway in the anterior–posterior or side-to-side directions [6,7]. Conventional posturography was not sensitive enough to detect the early stages of balance impairments [8,9]. Posture sway assessments can be improved by adding a more dynamic component involving weight shifting, after which the stability limit would be determined by pursuing active COP movements [10,11].

The age-related deterioration of balance control is reflected in less effective posture responses to balance perturbations. An inadequate postural reaction leads to a reduced capacity to recover from a postural perturbation during dynamic movement control. Hilliard et al. indicated that a considerable proportion of falls can be attributed to inadequate weight-shifting or stepping responses in older persons [12,13]. In community-dwelling fallers, posture sway control in the mediolateral direction was often disturbed by daily-life tasks [8,10]. The stability limit of each individual was challenged to facilitate dynamic movement control by the incremental increase in self-initiated perturbations. Maximum voluntary excursion (MVE) is defined as the range of movement where subjects preserve stance and do not take a step [14]. The study of the COP trajectory path can be used to estimate the border between static and dynamic corrective strategies for postural control. Previous studies found the range of voluntary COP excursion to decrease as the subjects’ age increased [15,16]. Based on the visual tracking of a moving COP target, a challenging approach would be developed to improve the ability to withstand posture perturbations within individual limits of postural stability.

Most clinical interventions often employed traditional modes of strength or balance training for older adults. Sometimes these exercises were not task specific and may not have been effective for improving particular aspects of dynamic control during functional activities [17]. Furthermore, the exercise intensity should be prescribed based on each individual’s performance level and movement capacity. The training volume and time ha to be adjusted as the individual progresses. The conventional programs could be repetitive and time-demanding. Previous studies recommended the use of computer assistive technology or the introduction of a game element to improve the subjects’ motivation for exercise participation. Some researchers indicated that the interactive gameplay would lead to more improvements in the dynamic balance control compared with a generic program [18,19].

A task-oriented approach was delivered via an interactive computer game paradigm. The intervention focused on voluntary movement involving a moderate challenge to the stability limit by introducing weight shifts. The gamebased programs involved repetitive multi-directional movements toward random target locations with varied speed, amplitude and precision. The engaging dynamic tasks emphasized reactive balance controls and interactions with the environment. This study developed an interactive balance evaluation and training platform. The system incorporated a computer game-based program with an instantaneous force plate evaluation. The training results of the experimental group were compared with an age-matched control group who kept usual activities only. Clinical balance and functional assessment scales were administered to these participants to validate whether interactive balance training could be successfully translated to their balance function and mobility.

## 2. Materials and Methods

### 2.1. Study Population

Twenty-nine community-dwelling individuals over the age of 65 years old and ten graduate students were recruited to participate in this study. The young students assisted in testing the whole procedure and just underwent a balance evaluation at baseline. The demographic characteristics of the subjects are summarized in Table 1. The experimental and control groups were comparable in terms of age, height and weight.

Upon arrival in the laboratory, all the participants were informed of the study procedures and signed an informed consent form. All experiments were performed with the approval of the University’s ethics committee on human research. Eligible older persons were able to walk independently in the community. Exclusion criteria based on their self-reported history and medical records included physical, visual, cognitive or any potential issues that would compromise their safe study participation.

None of the participant were taking any medications known to affect balance function. The elderly having a history of falls in the previous 12 months or receiving other exercise interventions at the same time were also excluded from this study. The elderly participants were randomly assigned to either an experimental group or a control group. The experimental group (*n* = 15) received three sessions of balance training per week for 6 weeks. Each session lasted 40 min including a warm up and rest periods as needed. The control group (*n* = 14) maintained daily activities as usual. Both groups underwent pre- and post-intervention testing.

### 2.2. Equipment of Computerized Balance Platform

**Figure 1:**
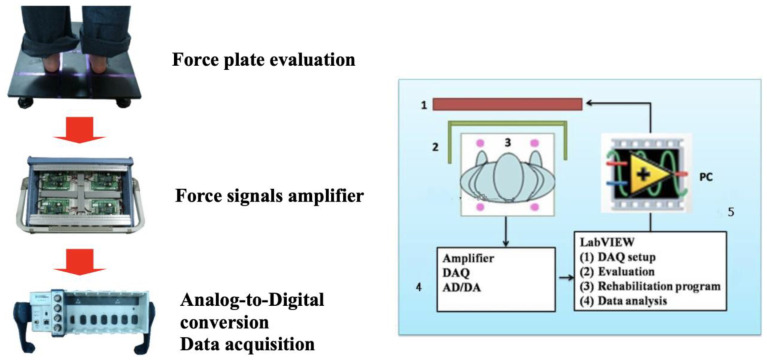
Experimental set-up.

The interactive balance system consisted of a computer device and a force platform. There were four uni-axial load cells (CRS03, Silicon Sensing, Devon, UK) installed on the bottom of the force plate. The vertical ground reaction forces were synchronously processed through a data acquisition device (cDAQ-9172, National Instruments, Austin, TX, USA) after a 16-bit analog-to-digital conversion at a sampling frequency of 100 Hz (Figure 1).

**Figure 2:**
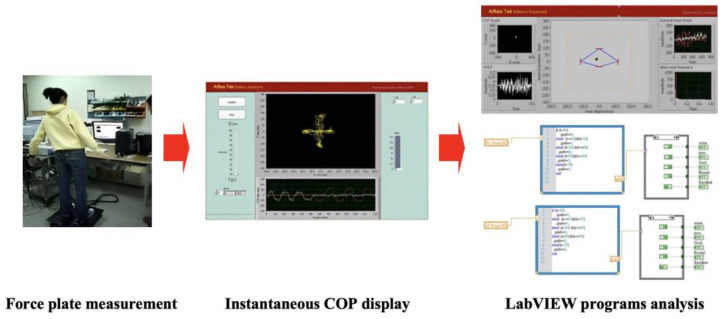
COP position in a time-series plot and subsequent analysis.

The COP signals were further analyzed using custom-made programs by LabVIEW 8.5 (National Instruments, USA). The position of the center of pressure was displayed as a time-series plot in the Cartesian coordinate system (Figure 2). The COP displacement was calculated by summing the actual distance between successive COP trajectories in the anteroposterior (AP) and mediolateral (ML) directions.

The participants were instructed to stand barefoot on the force plate and to remain with their body straight and arms loosely hanging by both sides. Their feet were kept in a fixed position 10 cm apart throughout all trials. For safety reasons, the investigator stood centrally behind the subjects and a walker was positioned in front throughout the whole procedure. The subjects were encouraged to shift their body weight as far as the limit of stability without altering foot position. The positions and displacement of the COP were acquired during three consecutive trials of maximal voluntary excursion in AP and ML directions. All three trials of weight-shifting movement were tested twice in each direction.

### 2.3. Video-Game-Based Balance Training

**Figure 3:**
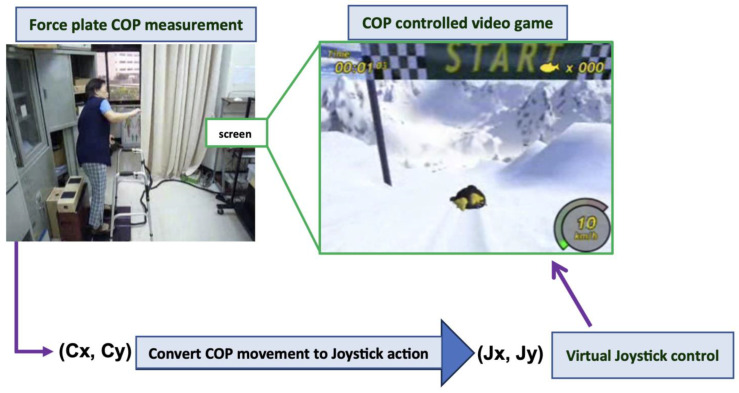
Game-based training framework.

An open source videogame was incorporated within the platform system (Figure 3). The visual feedback training was set to initiate once the player’s COP was moved. The COP was displayed as a penguin and was virtually converted using computer programming to a joystick control to guide the execution of the gameplay. The racing game showed that the penguin continuously advanced across a mountainside. The target position was to be controlled by weight-shifting action. Players needed to finish a series of courses within a specified time and score enough points. They needed to progress as soon as possible to gain points by collecting sufficient herrings scattered along the courses. The penguin was programmed to perform a tight turn or launch into the air to escape being crashed or reach required objects in various directions. The players could also reset the game when the penguin got stuck in any part of the course. The courses were composed of various terrain types that affected a racer’s performance. The players had to adjust their speed to allow for more maneuverability. The racers could lean forward to give the penguin some additional speed. Backward leaning stopped giving speed and, in turn, slowed it down. The participants could check the performance by viewing the scores and time after gameplay.

### 2.4. COP Signals Analysis

The ability to maintain postural control was measured by the parameters of COP displacement while the subjects reached maximal voluntary excursion. The evaluation of the maximal COP excursion was administered for each elderly before and after the 6-week gamebased training. The sway parameters were used to quantify the migration of the COP trajectory in terms of sway area, mean sway velocity, maximal sway range and total path length along the anterior–posterior and medial–lateral axis [20]. Our excursion task challenged the elderly to reach their stability limit, so the subjects with fair posture control could displace the COP quickly and more distally. An increase in the COP parameters would signify a more confident postural control. All parameters were computed for each trial and then averaged.

The sway path was defined as the excursion length of the COP trajectory within the BOS. The total path length was calculated by summing the actual distance between successive COP locations. The COP mean sway velocity was defined as the total distance travelled by the COP trajectory over time. It was calculated by dividing the sway path by the time of each trial’s duration. The COP sway area was calculated by computing the 95% confidence ellipse of COP anterior–posterior and medial–lateral coordinates [21].

### 2.5. Clinical Outcome Assessment

Functional postural control was assessed before and after 6 weeks of intervention by an assessor blinded to the grouping. Functional mobility tests were employed in this study, including Berg Balance Scale, Timed-Up-and-Go test (TUG) and functional reach test.

The Berg Balance Scale (BBS) was widely used as a functional balance assessment. The BBS consisted of 14 separate tasks ranging from sit-to-stand to one leg stance. The performance-based measures primarily evaluated transfers and static standing balance. The BBS items were rated on a 0 to 4 point scale based on performance quality, duration and assistance needed with a total score range of 0 to 56 points. The reliability and validity of this scale have been previously tested among elderly persons [5]. The cut-off scores for BBS ranged from 45 to 51 points according to former systematic reviews [22].

The Timed-Up-and-Go test was used to assess the mobility and the dynamic posture control. The participants had to get up from a 46 cm high chair with armrests, walk 3 m at normal pace, turn around, return to the chair and sit down. All the subjects completed the test by themselves and did not use any assistive devices. The TUG test measured the time the participants took to finish the test in seconds and the average of the three experimental trials was used in the analysis. The community-dwelling older adults requiring more than 13.5 s to complete the test were at higher risks for falling [23].

The functional reach test recorded the maximal distance that an individual can move their COM toward the boundary of the BOS. The subjects stood with their feet comfortably apart and reached forward as far as possible with shoulder flexion at 90 degrees without losing balance. The examiner measured the location of the third metacarpal using a yardstick attached to a wall, at about the acromion level, both before and after the reach, with the difference indicating their reach distance. Two practice and three recorded trials were completed, with the average being the overall performance. The functional reach distance to predict fall risks in healthy elderly ranged from 18 to 22 cm [24].

### 2.6. Statistical Analysis

The SPSS Statistics 21.0 (IBM Corporation, Armonk, NY, USA) was applied for data analysis. The Student *t*-test was used to analyze the differences for independent samples between groups at baseline. The COP sway parameters and clinical outcome measures in the experimental and control group were analyzed before and after the intervention by repeated measurement ANOVA. The differences were considered statistically significant when *p* < 0.05. The sample size was determined using the G*Power 3.13 software (Franz Faul University, Kiel, Germany). The investigators set the effect size to 0.45, with an alpha level of 5% and power of 80%. The calculation showed that the total sample size required to achieve sufficient power would be 26.

## 3. Results

The COP displacement parameters of the young and elderly subjects are shown in Table 2. The elderly revealed lower sway area (mm^2^), mean velocity (mm/s) and excursion range (mm) in all directions than the young participants (*p* < 0.05). The more significant differences were noted in the anterior–posterior axis rather than in the medial–lateral axis. The young and elderly groups revealed the most prominent differences in the COP sway area and maximal excursion range in the anterior direction.

As can be seen in Table 3, the experimental group experienced a significant increase in their COP sway area (mm^2^), mean velocity (mm/s) and excursion range (mm) in the medial–lateral direction over the training period. In the anterior–posterior direction, a significant improvement was also noted in the total path length and mean velocity, and the maximal excursion range was only increased in the anterior direction. The COP displacement parameters of the control group were not increased meaningfully over the same period.

The experimental group revealed a significant increase in the Berg Balance Scale over the training period (39.90 ± 5.80 vs. 49.45 ± 3.95 points, *p* = 0.0001). A similar improvement was also seen in the Timed-Up-and-Go test (17.08 ± 5.16 vs. 13.13 ± 3.39 s, *p* = 0.0020) and functional reach test (19.13 ± 5.34 vs. 23.81 ± 4.40 cm, *p* = 0.0011).

## 4. Discussion

The platform system was designed to investigate and improve the postural balance of the elderly. For the posture sway parameters, the inadequate control could be expressed as an increased or decreased COP excursion depending on the methodology used. Most previous studies asked participants to maintain a quiet stance in a steady state. Alhasan et al. [19,25] indicated that most falls among older adults were due to poor movement control when faced with perturbation. This system challenged the subjects to reach their stability limit as far as possible. The COP control was initially evaluated between the elderly and young people when responding to self-initiated perturbations. All the parameters of the COP dispersions were significantly different between the elderly and young persons. Gouglidis et al. indicated that the most prominent differences between the elderly and young people concerning COP control were mainly on the sagittal plane [26,27]. Our present finding was similar to the previous studies in that the elderly revealed a lower COP sway velocity and excursion range than the young people, especially in the anterior direction. Forward pivoting around the ankle imposed greater eccentric loading on the muscles than backward movement. The young participants could meet the activation requirement of the ankle to move more in the anterior direction by enhancing precise muscular control.

Rezaei et al. suggested that the ankle strategy controlled anterior–posterior movement, while medial–lateral stability was mainly controlled by the hip strategy [28]. The ankle’s muscular loading would be increased especially during weight shifting in the anterior–posterior direction. This study revealed that the reduced COP excursion in the sagittal plane could be related to the age-related decline in ankle movement control. Amiridis et al. concluded that older adults relied more on the hip muscles when responding to self-induced balance perturbations [29]. Increased risk of falling in the elderly has been associated with greater reliance on the hip mechanism when faced with external perturbation. The elderly had an impaired capacity to control the COP displacement especially in the anterior–posterior direction. This study helped to identify direction-specific posture control when responding to self-imposed perturbations. Balance training programs focusing on weight shifting in different directions could induce specific adaptations to the ankle or hip with loading and unloading mechanisms responsible for dynamic movement control.

This study was designed to enhance the dynamic posture sway of the elderly over the limits of stability. The game-based training involved a moderate challenge to balance control by introducing weight shifts toward various directions. Both anterior–posterior and medial–lateral balance performance was related to age and falling. The complete training program has to train the subjects to perform weight shifts in all directions [30]. This study hypothesized that our short-term program would have positive effects on all the COP variables. The experimental group revealed more COP displacement in the medial–lateral axis than in the anterior–posterior axis due to the nature of the game used. The computer programs related to ski slalom mainly involved medial–lateral movements to reach or escape objects. The racing game has the potential to induce more challenging movement in the medial–lateral direction. Eliciting anterior–posterior weight shifts was mainly for adjusting the moving speed. More experienced participants were familiar with the progress of computer games, which led to movements predominantly in the anterior direction. They would avoid posterior shifting to slow down, as observed in the present finding. The experimental group would cope with fast speed and move more quickly after training in each direction.

Amiridis et al. indicated that older adults could not only depend on the ankle strategy to counteract the gravitational torques imposed by extreme COG sway angles, but also on the hip strategy in order to return the COG within their safety limits [29]. When shifting a body sideways, the sidetoside movement was mainly controlled by the loading and unloading mechanism [31]. The activation requirements imposed on the hip muscles could be uncertain during ML weight shifting. Previous studies suggested that weight shifting in the A/P direction would increase the contribution of the ankle mechanism in static postural control through enhancing the active control of ankle stiffness [27,32]. Previous findings concluded that posture sway control would be improved by strengthening the ankle dorsiflexors in the elderly [33]. The plantar and dorsi-flexors have been known to have a primary role in quiet stance control. This study applied game-based training to challenge the participants to move rapidly toward the border of the BOS in all directions. Visually driven weight shifts would facilitate feedforward preparatory control. The limit of stability could be approached through pivoting around the ankle. The extreme ankle movement would impose further muscular loading and proprioceptive feedback to deal with balance perturbation. Our findings supported the notion by Sihvonen et al. [34] that visual feedback training can help improve postural control and functional mobility.

Winter et al. indicated that the ankle and hip mechanisms did not work independently to control M/L and A/P movement sway [31]. The tibialis anterior and soleus muscles also acted as ankle invertors, and they would contribute to the control of the M/L postural sway. Our prescribed training improved the COP control mainly in the medial–lateral direction due to the nature of the ski-related game used. It should be noted that a significant increase was also found in the total displacement path and mean velocity after training in the A/P axis. This study suggested that the M/L predominant weight shifts did not only improve the M/L sway control but also the A/P movement. In addition to the loading and unloading movement control, the secondary ankle strategy as proposed by Gatev et al. [32] would develop to deal with the M/L predominant perturbation. The side-to-side movement training could be shifted from the hip to the ankle through strengthening the ankle mechanism. The increase in the total anteroposterior sway path and velocity could be an early sign of the balance improvement by promoting the use of the ankle strategy. Our findings supported the collaborative contribution of the ankle and hip mechanisms to the control of multi-directional equilibrium. To challenge an individual’s stability limit, one could reorganize the relative contributions of different postural strategies to balance their control.

When reviewing the literature regarding game-based training, most studies recommended the use of commercialized off-the-shelf games [3,19,30]. The progress would be followed up approximately, based on the performance time and total score after gameplay. This study incorporated interactive training into force platform evaluation. An elaborate and expensive virtual display apparatus was not required in our system. The training volume could be adjusted by altering game settings according to an instantaneous COP analysis. The customized program ensured an appropriate level of challenge to each individual’s stability limit and the elderly could conform to the prescribed training relative to their own movement capacity. Individually tailored practices and close monitoring of training effects were essential to the continuous progression in the elderly.

The reduced capacity to control the COP displacement over the BOS boundary might increase the fall risks, particularly during the performance of daily activities that challenge the stability limits [35]. This study recommended the use of computerized training as an effective way to improve the COP movement control in healthy elderly people. A similar improvement was also seen in functional mobility tests. Previous studies found the functional assessments to have a ceiling effect, which would be evident in healthy older adults [4,5]. Although the cut-off scores for the tests were not consistent among studies, the improvement in the functional mobility scores could be interpreted by the British Geriatrics Society as an improvement in frailty status [36]. This study suggested that the short-term programs could promote functional improvement as well. The control of postural movement was enhanced in this study, and this ability should be essential to functional independence in the elderly.

The limitation of this study was that the relatively small sample size would limit the generalizability of these results to the overall geriatric population. This study did not compare our training system to other forms of interventions. The pilot study only dealt with a comparison between the treatment and non-treatment group. These findings just ensured that the improvement in the healthy elderly was from the prescribed training itself. The elderly should maintain static posture control as well. Quiet stance recording would also be needed to test if our dynamic training has any effects on static balance control. A longer period of training with extensive follow-up is required to confirm whether the immediate effect of the prescribed training is retained in future studies.

## 5. Conclusions

This study revealed the immediate effects of interactive training programs on the older adults living in the community. The elderly participants improved their ability to control the center of pressure more quickly and precisely by tracing a COP-controlled target used in the game-based platform. The stability limit of each individual was challenged to facilitate dynamic posture control by the incremental increase in self-initiated perturbations. The platform system could assist with the transferring of the training effects into daily functional mobility in the elderly.

## Figures and Tables

**Table 1:** Summary of the participants’ demographic characteristics at baseline.

	Young	Experimental Old	Control Old
Number	10	15	14
Male/Female	6/4	9/6	8/6
Age (years)	24.25 ± 1.425	78.00 ± 7.028	79.90 ± 5.539
Height (cm)	166.93 ± 8.84	155.20 ± 8.51	156.17 ± 7.54
Weight (kg)	63.89 ± 16.97	54.57 ± 11.15	54.95 ± 10.79

Data are presented as mean ± SD.

**Table 2:** Summary of the COP path range (mm), sway velocity (mm/s) and area (mm^2^) at baseline.

	Young (*n* = 10)	Elderly		*p* Value
		Control (*n* = 14)	Experimental (*n* = 15)	
M/L total path	2530.42 ± 762.68	1609.97 ± 385.45	1413.31 ± 423.87	0.0025 *
A/P total path	2857.69 ± 520.12	1787.16 ± 441.68	1699.87 ± 509.24	0.0002 *
Total path	4528.19 ± 988.28	2764.66 ± 564.11	2645.59 ± 711.54	0.0005 *
M/L sway range	264.55 ± 17.58	181.97 ± 45.52	166.00 ± 60.95	0.0000 *
A/P sway range	186.87 ± 13.13	93.62 ± 19.83	96.72 ± 37.77	0.0000 *
Max range to R	130.30 ± 10.46	99.29 ± 25.25	81.52 ± 30.09	0.0000 *
Max range to L	134.24 ± 8.69	82.67 ± 30.06	84.47 ± 33.77	0.0000 *
Max range to A	81.26 ± 11.73	39.02 ± 16.46	49.62 ± 24.31	0.0000 *
Max range to P	105.61 ± 12.11	56.60 ± 24.01	39.86 ± 26.64	0.0000 *
M/L mean velocity	63.44 ± 20.72	35.62 ± 43.88	40.62 ± 33.88	0.0048 *
A/P mean velocity	68.13 ± 21.62	28.29 ± 28.25	39.03 ± 26.48	0.0005 *
Mean velocity	98.88 ± 36.62	53.58 ± 62.36	59.81 ± 38.69	0.0010 *
COP sway area	24658.91 ± 1607.95	8518.03 ± 2482.06	8942.27 ± 6825.78	0.0000 *

Data are presented as mean ± SD. * *p* < 0.05.

**Table 3:** Comparison between pre/post-intervention in the COP path range (mm) and velocity (mm/s).

	Control Group (*n* = 14)			Experimental Group (*n* = 15)		
	Pre-Intervention	Post-Intervention	*p* Value	Pre-Intervention	Post-Intervention	*p* Value
M/L total path	1609.97 ± 385.45	1578.88 ± 629.88	0.8564	1413.31 ± 423.87	1840.87 ± 492.46	0.0101 *
A/P total path	1787.16 ± 441.68	1828.24 ± 373.94	0.7315	1699.87 ± 509.24	2562.91 ± 532.52	0.0002 *
Total path	2764.66 ± 564.11	2769.48 ± 722.43	0.9810	2645.59 ± 711.54	3564.39 ± 773.93	0.0027 *
M/L sway range	181.97 ± 45.52	190.89 ± 40.88	0.4446	166.00 ± 60.95	241.58 ± 50.42	0.0002 *
A/P sway range	93.62 ± 19.83	101.56 ± 28.85	0.2776	96.72 ± 37.77	113.25 ± 34.35	0.1538
Max range to R	99.29 ± 25.25	103.39 ± 19.29	0.4471	81.52 ± 30.09	123.65 ± 26.39	0.0000 *
Max range to L	82.67 ± 30.06	87.50 ± 29.36	0.5584	84.47 ± 33.77	117.93 ± 26.99	0.0000 *
Max range to A	39.02 ± 16.46	41.27 ± 22.17	0.2896	49.62 ± 24.31	69.90 ± 19.19	0.0192 *
Max range to P	56.60 ± 24.01	55.29 ± 19.94	0.8262	39.86 ± 26.64	49.59 ± 19.48	0.1061
M/L mean velocity	35.62 ± 43.88	40.07 ± 54.90	0.3546	40.62 ± 33.88	65.07 ± 54.90	0.0292 *
A/P mean velocity	28.29 ± 28.25	32.18 ± 32.49	0.2864	39.03 ± 26.48	61.40 ± 28.97	0.0381 *
Mean velocity	53.58 ± 62.36	55.39 ± 44.91	0.2535	59.81 ± 38.69	93.39 ± 44.91	0.0333 *
COP sway area	8518.03 ± 2482.06	9846.44 ± 4025.16	0.1965	8942.27 ± 6825.78	13581.98 ± 6716.52	0.0216 *

Data are presented as mean ± SD. * *p* < 0.05.

## Data Availability

All relevant data are within the manuscript.
